# Comparison between biparietal bipolar and uniparietal bipolar radio frequency ablation techniques in a simultaneous procedural setting

**DOI:** 10.1007/s10840-020-00852-5

**Published:** 2020-08-24

**Authors:** Francesco Matteucci, Bart Maesen, Carlo De Asmundis, Elham Bidar, Gianmarco Parise, Jos G. Maessen, Mark La Meir, Sandro Gelsomino

**Affiliations:** 1 Cardiothoracic Department, Maastricht University Hospital, Maastricht, The Netherlands; 2 Department of Cardiothoracic Surgery, Cardiovascular Research Institute Maastricht University, Universiteitssingel 50, 6229 ER Maastricht, The Netherlands; 3 Cardiothoracic Department, Brussels University Hospital, Brussels, Belgium

**Keywords:** Biparietal bipolar ablation, Bipolar radio frequency energy, Bipolar prototype, *In vitro* bipolar ablation

## Abstract

**Purpose:**

To make an *in vitro* evaluation of the lesion size and depth produced in two different sets of radio frequency energy bipolar delivery: simultaneous biparietal bipolar (SBB) and simultaneous uniparietal bipolar (SUB).

**Methods:**

Two separate prototypes have been built for our purpose: one to be used in SBB mode and the other to be used SUB mode. Forty left atrium samples were taken from the hearts of freshly slaughtered pigs. They were ablated into a simulator ABLABOX, where blood flow, temperature, and contact force were controlled. After being sliced into a cryotome, the samples were digitalized by a flatbed scanner, and the images were analyzed by a computer morphometric software.

**Results:**

Transmural lesions were achieved in 18/20 samples (90%) in SBB, while SUB showed transmurality in 9/20 samples (45%). Overall maximum diameter (*D*_MAX_) resulted larger in SUB than in SBB (2.43 ± 0.30 mm, 1.62 ± 0.14 mm, respectively; *p* < 0.05): Moreover, maximum epicardial and endocardial diameters (*D*_EPI_ and *D*_ENDO_, respectively) were wider in SUB group than SBB group (2.28 ± 0.30 mm, 2.26 ± 0.40 and 1.60 ± 0.14 mm, 1.59 ± 0.15 mm, respectively; *p* < 0.05). We observed the same tendency in lesion depth: The total area and volume (*A*_TOT_ and *V*_TOT_) were broader in SUB group than in SBB one (581.01 ± 65.38 mm/mm^2^, 58.10 ± 6.53 mm/mm^3^ and 521.97 ± 73.05 mm/mm^2^, 52.19 ± 7.30 mm/mm^3^. respectively; *p* < 0.05).

**Conclusions:**

In contrast with the smaller lesion sizes, the biparietal bipolar group showed a higher transmurality rate. These findings may suggest a better drive of the energy flow when compared with SUB lesions.

## Introduction

Hybrid ablation (HA) exploits the concomitant effect of epicardial and endocardial procedures performed together by a surgeon and an electrophysiologist. It combines epicardial ablation performed by bilateral thoracotomy to a standard transcatheter endocardial ablation. Apart from joining the advantages of the two procedures, this technique allows the electrophysiologist to test the ablation, to add touch-up ablation if necessary and to reach part of the heart that cannot be ablated by the surgeons [1].

Radio frequency (RF) is the energy source mainly employed, and RF tools are based on unipolar or bipolar technology. Moreover, the endocardium lesions are obtained through the use of unipolar unidirectional punctual catheters, while epicardial ones are achieved through the use of bipolar unidirectional RF implemented in different tools. The goal of all the techniques employed in the treatment of atrial fibrillation (AF) is the achievement of transmural lesions that are considered the indispensable requirement for durable AF-free conditions [2, 3]. HA significantly improved 1-year follow-up in patients [4], but the results are not still acceptable in terms of the rate of freedom from recurrence, particularly in long-standing persistent AF [5].

Nonetheless, bipolar tools used in the hybrid ablation setting may not exploit the entire potential of this new technique, and often, the tissue penetration is limited only to a thin shallow rim [6]. We hypothesize that the settlement of the tissue interposed between the ablating poles (biparietal configuration) may result in a higher rate of transmural lesions. The objective of the present study is to compare *in vitro* a biparietal bipolar prototype to a uniparietal bipolar catheter in a simultaneous ablation setting.

## Material and methods

### The *in vitro* simulator

**Fig. 1:**
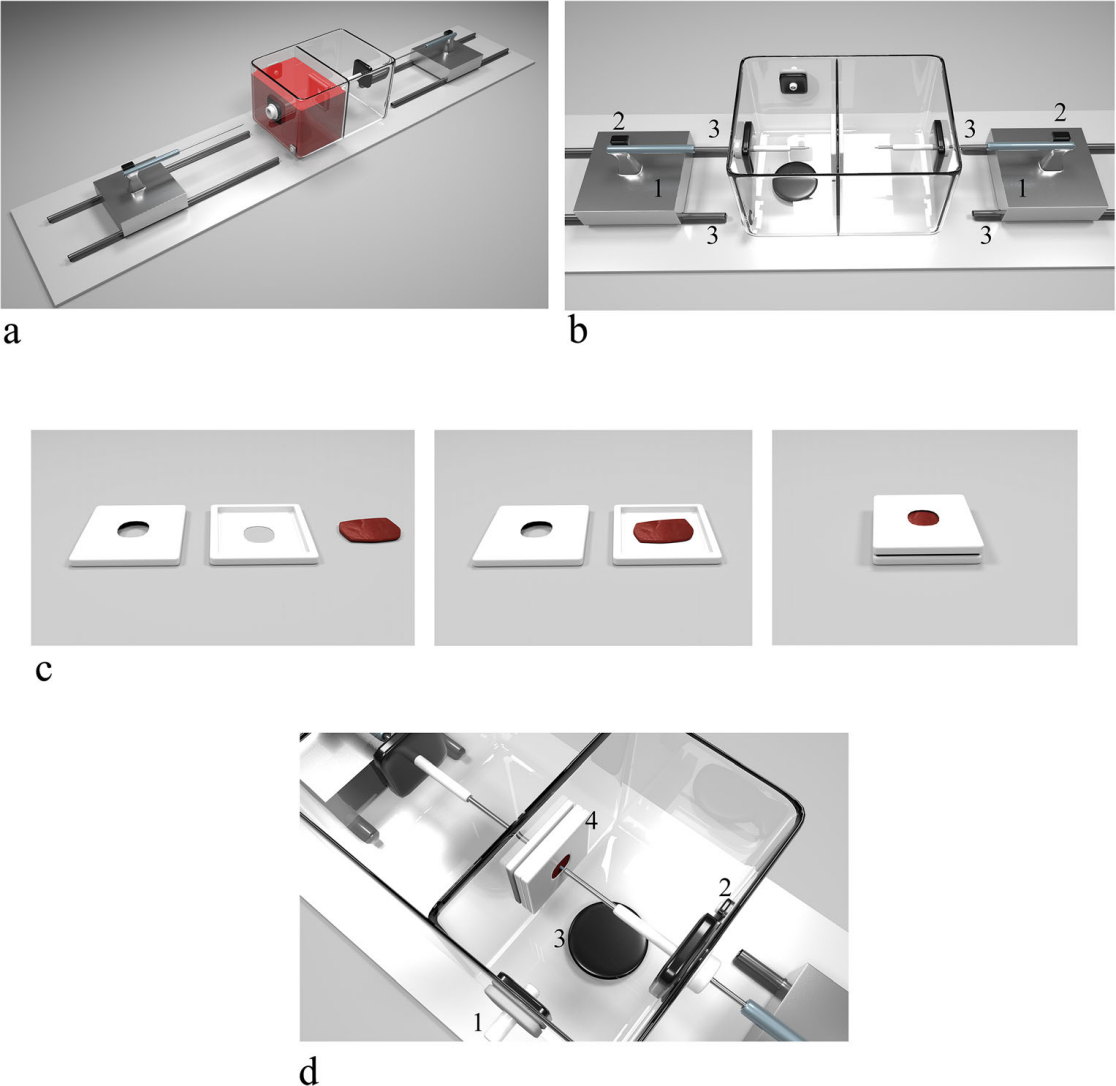
**a** The ABLABOX overview. **b** Particular of the ablation area: (1) catheter holding system, with pressure sensor (2). The holder is free to move over a couple of rails (3) with an adjustable stop system. **c** Placement of the sample into the simulator: the sample is placed between two plastic holding supports, both with a central open to let the tips of the catheters facing the tissue**.** The closure is obtained by the use of neodymium magnets embedded into the plastic frame. **d** Endocardial mimicking chamber: (1) blood flow pump inlet, (2) outlet, (3) adjustable magnetic stirrer motor, (4) magnetic locking tissue sample holder

For the present study, we used a simulator (ABLABOX, IDEE—Instrument Development Engineering and Evaluation, Maastricht University, NL), previously described (Fig. 1) [7].

### The prototypes

#### Biparietal bipolar

**Fig. 2:**
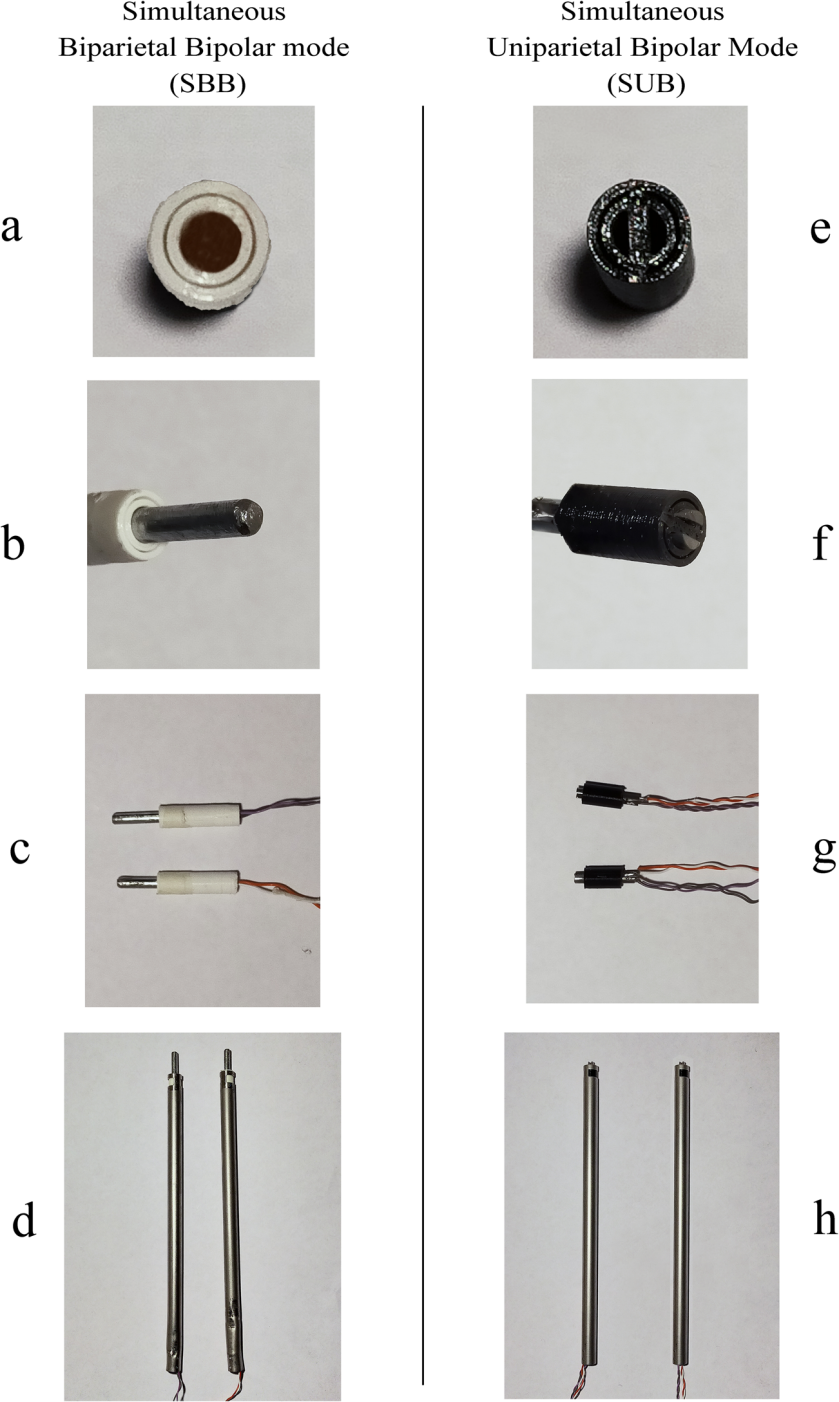
Catheter assembly sequences in SBB (simultaneous biparietal bipolar) setting and SUB (simultaneous uniparietal bipolar) setting. (a) 3D printed supports, (b) electrode placement into the supports, (c) electric connections, and (d) placement of the system into an aluminum handle ready to be mounted into the simulator, (e) 3D printed supports for uniparietal technique, (f) electrodes placement into the support, (g) electric wiring connections, final assembly into aluminum handle

A 3D printed polylactic acid (PLA) cylindrical (30 mm × 0.80 mm external diameter) electrode supports have been printed (Fig. 2(a)). A 2.5-cm length × 0.4 mm in diameter cylindrical metal rods have been placed inside the holders, working as the electrodes (Fig. 2(b, c)). The whole package, including the electric connections, has been included in a hollow aluminum cylinder (20 cm length × 0.95 cm external diameter) (Fig. 2 (d)) to be mounted in the simulator during the experiment.

#### Uniparietal bipolar

The prototype for the uniparietal bipolar technique has the same external shape and dimensions, except the 3D printed electrode support that is shaped to embrace and leave electrically isolated a couple of electrodes working in bipolar uniparietal way (Fig. 2 (e)**)**. Indeed, the same 4-mm metal rod has been cut on its length with a 1.2-mm-thick cutting disc to respect the external dimensions of the electrode of the biparietal bipolar catheter (Fig. 2 (f)). Likewise, the holder and the electrodes, together with their electric connections, are included in the same shape hollow cylinder of the previously described catheter (Fig. 2 (g, h)). The prototypes were both connected to a standard bipolar radio frequency generator.

### Samples

**Fig. 3:**
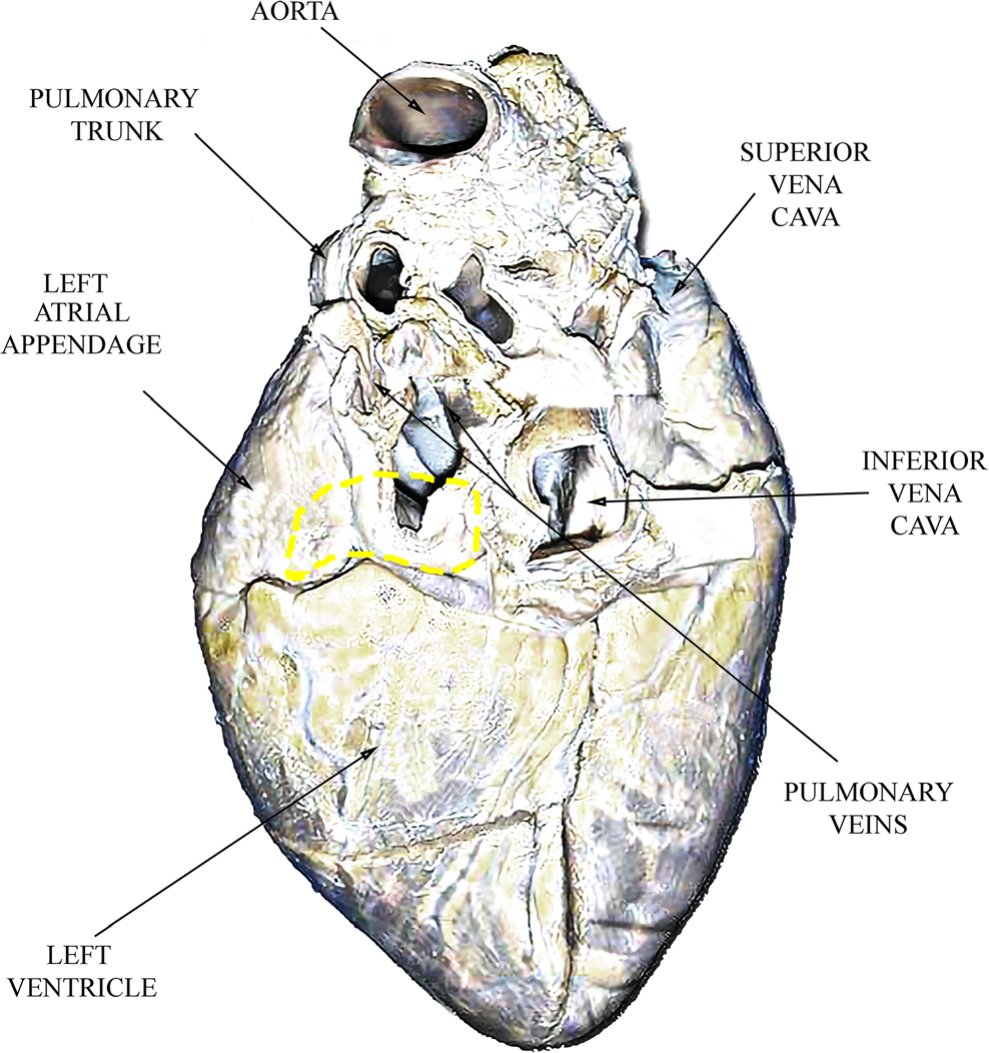
Pig’s heart posterior wall. The yellow dotted area depicts the origin of the samples

Samples came from freshly slaughtered pigs, and left atria were isolated to be employed in the present study. Concerning the sections done by the personal of the company while slaughtering the animals, the lesions were performed in the left atrium inferior posterior wall, on the broad junction (difference of species with human left atrium) [8], to the pulmonary venous compartment avoiding when possible the pectinate muscles (Fig. 3).

**Fig. 4:**
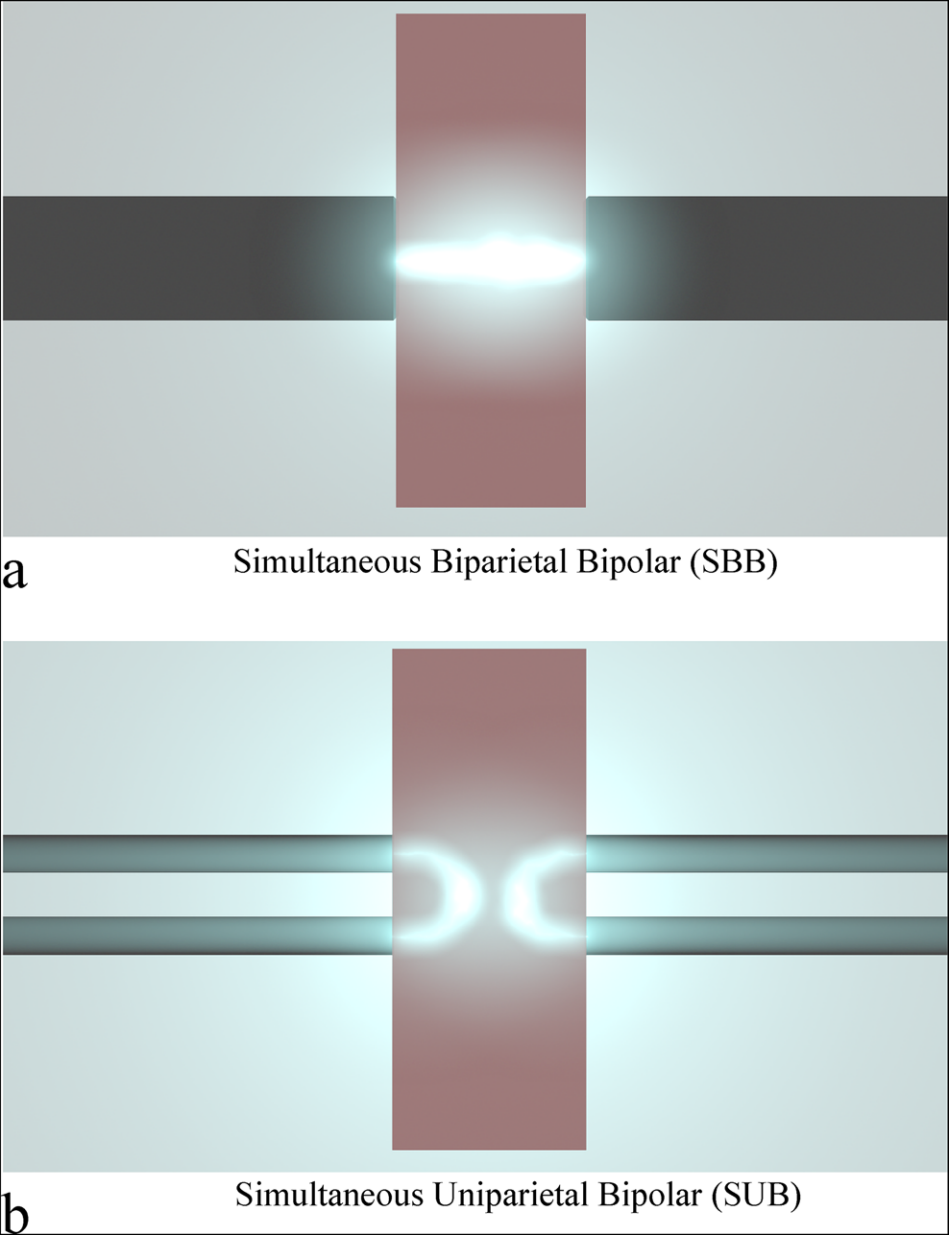
Scheme of energy spread within the tissue. **a** The energy crosses the tissue along the shortest distance between the electrodes. **b** The energy creates an arch from the two poles

After being washed to remove the residual blood with NaCl (B. Braun, Melsungen, Hessen, Germany), they were put into a transport medium RPMI 1640 (Sigma-Aldrich, St. Louis, MO, USA). Samples were randomly assigned to two groups of study: (1) simultaneous biparietal bipolar (SBB), where the poles were placed on the opposite sides of the sample and the radio frequency crossing the sample width, and (2) simultaneous uniparietal bipolar (SUB) mode, where the electrodes were placed on only a side of the sample (Fig. 4). Twenty samples of each group were employed for a total of 40 samples.

### Experimental setup

The power console had a fixed RF output (28.5 W at 114 Ω load) that was kept the same for both groups. The control of the delivery and amount of RF was controlled by an internal dynamic monitoring algorithm checking the impedance and stopping the energy delivery after transmurality was reached. In the SBB group, we used two identical consoles for the simultaneous RF application. The simulator settings were fixed in 5 l/min of blood flow to mimic the human cardiac output [9], contact forces of 300 g each side, temperature at 38 °C, and stirrer motor at 400 RPM.

### Morphometric evaluation of myocardial tissue ablation

The samples harvested were put in 10% neutral buffered formalin for 2 days. Hereafter, the specimens were included in the Tissue-Tek® OCT™ medium and sliced by a Leica CM 3050 cryostat (Leica Biosystems, Wetzlar, Germany) at − 20 °C. The thickness of every single slice was fixed to 0.1 mm. Furthermore, the slices so obtained, were laid down on a transparent acetate sheet, left drying at room temperature, and covered with another sheet. Hereafter, a flatbed scanner (Perfection V39 Scanner, Epson America, Inc., 3840 Kilroy Airport Way, Long Beach, CA 90806) was employed for scanning the sheets at 600 dpi of resolution. Furthermore, the digitalized images were imported in an image processing software (ImageJ version 1.48 software; National Institutes of Health, Bethesda, MD, USA) to be measured. In each sample, the following parameters were calculated: the maximum diameter (*D*_MAX_), meaning the largest diameter measure within the whole stack of slices, and the maximum epicardial and endocardial diameters (*D*_EPI_ and *D*_ENDO_, respectively), indicating the largest measure of the diameter within endocardial and epicardial sides. Also, the total area (*A*_TOT_), calculated as the sum of the ablated area of each tissue slice, was determined. The total volume (*V*_TOT_) was calculated by multiplying the total area (*A*_TOT_) by its fixed thickness value setup in the cryostat (0.1 mm) (Table 1).

**Table 1:** Parameters calculated

Parameter	Ablation modalities		
	SBB	SUB	*p* value
Sample thickness (mm)	4.36 ± 0.20	4.20 ± 0.20	> 0.05
Transmurality (%)	90	45	-
*D*_MAX_ (mm)	7.06 ± 0.62	10.21 ± 1.26*	< 0.05
Indexed value	1.62 ± 0.14	2.43 ± 0.30*	
*A*_TOT_ (mm/mm^2^)	2275.79 ± 318.50	2440.27 ± 274.61	0.08
Indexed value	521.97 ± 73.05	581.01 ± 65.38*	0.01
*V*_TOT_ (mm/mm^3^)	227.57 ± 31.85	244.02 ± 27.46	0.08
Indexed value	52.19 ± 7.30	58.10 ± 6.53*	0.01
*D*_EPI_ (mm)	7.00 ± 0.63	9.58 ± 1.28*	< 0.05
Indexed value	1.60 ± 0.14	2.28 ± 0.30*	
*D*_ENDO_ (mm)	6.93 ± 0.65	9.52 ± 1.71*	< 0.05
Indexed value	1.59 ± 0.15	2.26 ± 0.40*	

Data are shown as mean ± standard deviation or number (percentage) as appropriate

*Abbreviations*: *D*_*MAX*_ total maximum diameter, *A*_*TOT*_ total area, *V*_*TOT*_ total volume, *D*_*EPI*_ maximum diameter at the epicardial side, *D*_*ENDO*_ maximum diameter at the endocardial side

*Significant SBB vs. SUB

The replicability and objectiveness assessment of the measurements was completed by interobserver (GP) and intraobserver evaluation (FM).

### Statistical analysis

The distribution of data was evaluated by using the Shapiro-Wilk normality test (StatsDirect Ltd., 9 Mountwood Road, Birkenhead, Merseyside CH42 8NQ, UK). If from normal distribution, data were expressed as mean plus/minus standard deviation. If not, the median (lower-upper quartile) was expressed. The comparison of the measurements of the groups was tested through the unpaired *t* test or Mann-Whitney test. The measurements were indexed by the thickness (average) of all the samples. Linear dimensions (*D*_MAX_, *D*_EPI_, *D*_ENDO_) were expressed in millimeters per millimeter, while surface (*A*_TOT_) and volume (*V*_TOT_) dimensions were expressed as square millimeters per millimeter and cubic millimeters per millimeter. The intraobserver (FM) and interobserver variabilities (GP) were tested for *A*_TOT_ and *D*_EPI_ in ten casually sorted specimen using *κ*-statistics. The value stands between *κ* 0 (no agreement) and 1 (perfect agreement).

## Results

**Fig. 5:**
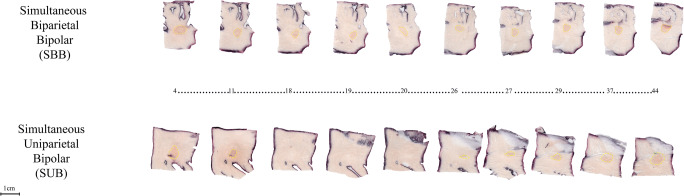
Progressive slice sequence. Ablation lesion formation progression while slicing the samples perpendicular to the direction of the catheters. The numbers show the lesion shape and dimensions in the study groups at that particular number of the slice pack. The SBB group shows a continuity of the lesions throughout the thickness of the sample. The SUB group shows a discontinuity in the progression of the lesion

No audible steam pops were encountered during the procedure, and no perforations were observed. The slices obtained formed a progressive sequence where modification of the shape of the lesion was appreciated (Fig. 5).

**Fig. 6:**
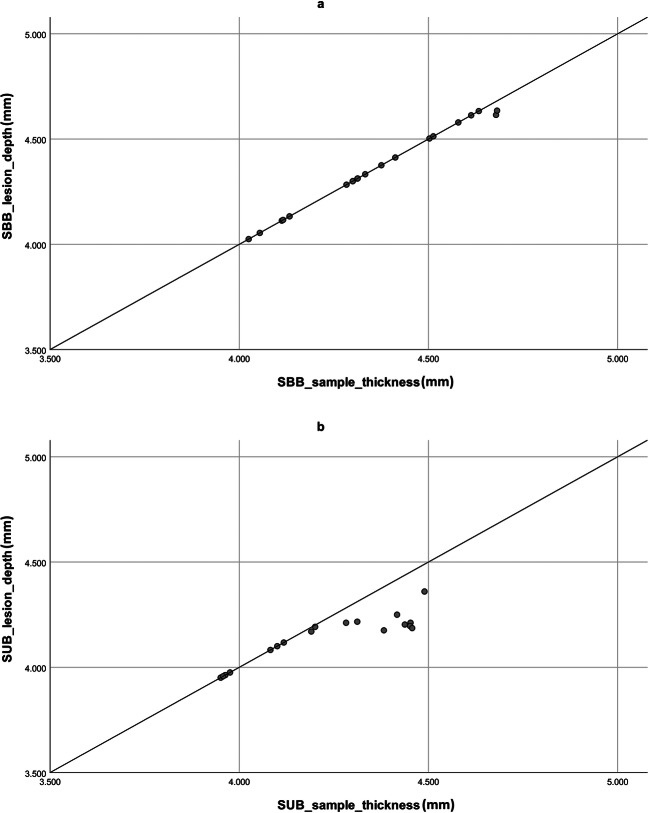
Scatter plot. Lesion depth is plotted against tissue thickness. Transmural lesions fall on the identity line. **a** Simultaneous biparietal bipolar. **b** Simultaneous uniparietal bipolar

Both the intra- and interobserver variabilities for the measurement of *D*_EPI_ (*k* = 0.97, *k* = 0.91, respectively) and *A*_TOT_ (*k* = 0.95, *k* = 0.89, respectively) were low, indicating an excellent repeatability and reproducibility of the measurements. Samples showed a thickness of 4.36 ± 0.20 mm in SBB and 4.20 ± 0.20 mm in SUB, with no significant difference (*p* > 0.05). Transmural lesions were detected in 18 samples over 20 (90%) in SBB group, while in SUB, transmural lesions were present in 9 samples over 20 (45%) (Fig. 6).

The overall *D*_MAX_ was significantly smaller in SBB than in SUB (1.62 ± 0.14 mm and 2.43 ± 0.30 mm, respectively; *p* < 0.05). Moreover, the *D*_EPI_ and *D*_ENDO_ in SBB group (1.60 ± 0.14 mm and 1.59 ± 0.15, respectively) were smaller than *D*_EPI_ and *D*_ENDO_ of SUB (2.28 ± 0.30 and 2.26 ± 0.40, respectively; all *p* < 0.05).

The *A*_TOT_ in SBB was smaller than *A*_TOT_ in SUB (521.97 ± 73.05 mm/mm^2^ and 581.01 ± 65.38 mm/mm^2^, respectively; *p* < 0.05). Likewise, the *V*_TOT_ was smaller in SBB compared with SUB (52.19 ± 7.30 mm/mm^3^ and 58.10 ± 6.53 mm/mm^3^, respectively; *p* < 0.05).

## Discussion

In the present study, we tested *in vitro* two different approaches of bipolar application: simultaneous biparietal bipolar and uniparietal bipolar settings. The results we obtained show a high rate of transmurality in the biparietal bipolar group and smaller sizes of the lesions induced in comparison with the uniparietal bipolar group. These aspects may suggest that the RF spreads broad mainly over the first few layers of the tissue, but it does not penetrate deeply. Only the bipolar biparietal setting seems to ensure a better propagation along the whole thickness of the tissue.

The difference in unipolar and bipolar settings resides in the different placements of the electric poles. Indeed, in unipolar mode, the energy flows from an emitter placed at the top of the catheter and an indifferent (ground) pole represented by the large electrode placed generally on the back of the patient. Contrarily, in bipolar mode, the positive and negative poles are placed side by side at the end of the catheter. In uniparietal setting, both unipolar and bipolar settings provide the poles to be displaced only from one side of the tissue (radio frequency catheter ablation or epicardial tools). In contrast, the biparietal mode provides the electrodes facing the tissue from the opposite side, resulting in the tissue being interposed between the poles (biparietal bipolar clamp).

Also, the behavior of the energy differs significantly. Indeed, in unipolar mode, RF spreads centrifugally from the emitter to the indifferent pole, while in bipolar technique, the RF diffuses from the close poles placed on the top of the catheter [10, 11].

From the clinical point of view, most ablation procedures are performed through catheter ablation, and apart from the use of cryogenic energy for ablating the pulmonary veins in the early onset of paroxysmal AF [12], RF energy is widely used for the treatment of AF. The application of RF catheter ablation is limited to unipolar uniparietal delivery. In contrast, in the epicardial thoracoscopic approach, cardiac surgeons can use both uniparietal and biparietal bipolar settings. The goal of all the ablation procedures is the achievement of transmural lesions [3, 13] leading to electric block. Remarkably, lesion gaps, even smaller than 1 mm, either lead to an incomplete electric isolation of the atrium or represent themselves a pro-arrhythmic element [14, 15].

The bipolar setting in ablation procedures seems to offer a higher rate of transmurality [16, 17] in comparison with unipolar technique. Especially, the use of biparietal bipolar clamp in either open chest surgery or mini-invasive thoracoscopic approach seems to represent the gold standard in AF, creating transmural, long-lasting, and reliable lesions [3, 18]. Another important aspect is represented by the lacking of the cooling circulating blood flow effect. Indeed, in bipolar setting, the electrodes are side by side and very closed to each other. Thus, the energy flow results in a very focused and narrow path between the poles. The significant consequence is a faster, useful ablation temperature reach with reducing over thermal risks of neighbor tissues [16].

Nonetheless, it must be addressed that bipolar tools used in the clinic, except biparietal bipolar clamp, do not include the interposition of the tissue between the electrodes. Remarkably, the biparietal setting in the bipolar clamps seems to represent the fastest and reliable tool to obtain transmural lesions in open-chest procedure [3, 19, 20]. The use of the clamp in this latter procedure provides a cardiopulmonary bypass followed by the insertion of one jaw of the clamp inside the atrium, thus a biparietal bipolar setting. Nonetheless, while with this setup, the clamp is a true biparietal; when it is employed only epicardically, it surrounds the pulmonary veins, interposing only the epicardium between the jaws. Therefore, this setting cannot be considered as genuinely biparietal.

The findings of the present study confirm that the biparietal bipolar setting shows a higher transmural lesions rate in comparison with the uniparietal technique. Contrarily, compared with the biparietal setting, the linear dimension of the lesions obtained (*D*_MAX_, *D*_EPI_, *D*_ENDO_) resulted broader in the uniparietal group. Likewise, the lesion depth (*A*_TOT_, *V*_TOT_) resulted wider in uniparietal setting than the biparietal one. These results may be clarified with the evidence that the RF current density is inversely proportional to the square of the distance between the electrodes, and the density is higher along the shortest distance between the electrodes [21–23]. As a consequence, the closeness of the poles when in the biparietal setting may result in a better spread through the wall tissue instead of uniparietal bipolar mode. Likewise, the same results may suggest that the diffusion of the energy in the uniparietal bipolar setting group is more randomly driven, causing a broader lesion in size and depth, but not reaching high transmurality.

However, our findings confirm that for tissue thickness lower than 4.2 mm, the result with the SUB is comparable with SBB. Indeed, these devices are widely used in clinical practice. Nonetheless, multiple simultaneous applications, long ablation times, and high contact force pressures are necessary to reach reliable lesions [24, 25].

Since AF patients have been reported to have thinner atrial walls, tissue perforation might be an issue when a transmural lesion is obtained. We have neither observed signs of damage macroscopically (carbonization) nor have we experienced steam pops. It has been previously demonstrated in ex vivo experiments that the perforation forces are higher in atria compared with swine [26], and the contact forces employed by these authors forces were, however, significantly lower than those applied in our experiments. Therefore, this might suggest that our setup was safe. Nonetheless, this warrants further research.

### Clinical repercussions

The two-stage hybrid results in an AF freedom of 56% without the use of anti-arrhythmic drugs [27]. Experts agree that new, improved tools are necessary to a complete isolation o the left atrium [28]. One of the main drawbacks of the tools in use is the incompleteness of linear lesion leading to gaps and failing a complete LA isolation [19]. With the magnetic coupling and with a biparietal design, it would be possible to make a complete one-stage LA isolation. The magnetic force should also allow to squeeze the tissue between the poles theoretically, making the thickness of the atrial wall and the epicardial fat to not influence the effectiveness of the lesion.

Long-lasting transmural PV isolation is obtained with bipolar clamps that use bipolar bidirectional radio frequency [20], and our approach implements the use of such bipolar bidirectional clamps that can be offered to patients with or long-standing persistent AF that is more difficult to treat. Our findings suggest that bipolar biparietal setting might represent a promising technique to achieve reliable endo-epicardial lesions. A key issue to obtain optimal results is the perfect alignment and stability of the poles, and the use of (electro)magnetism might represent a possible solution.

The clinical application of true biparietal bipolar HA is far for being established. Research on a custom-made bipolar HA prototype is ongoing, keeping into consideration different approach strategies.

### Limitations

This study has some limitations that need to be mentioned. First, we used ex vivo tissue, and the absence of microcirculation, acting like a heat sink, could have led to an overestimation of our results. Second, the RF generator used in the study has not been built to be employed in the biparietal bipolar setting. Hence, the property algorithm of the manufacturer controlling RF delivery could have driven higher/lower output based on registered impedance, and so in the reading of our findings, it must be taken into account. Third, the prototype tips had a different shape from the catheters commonly used for this purpose. This evidence, due to the fixed energy output of the generator, could have led to a decreased/increased power distribution along the electrodes. Fourth, we evaluated the necrosis only from a visual observation; we did not include a histological evaluation such as the TUNEL (terminal deoxynucleotidyl transferase [TdT] dUTP nick end labeling) because of the number of layers produced. Fifth, we did not measure the temperatures reached during the ablation. This might have given more insights since the physics of lesion formation between the catheters can be different.

Finally, the tissue employed for this study came from 1-year-old healthy pig breed for human consumption. Clearly, these hearts did not show pathological conditions typical of AF, like the fibrosis development.

## Conclusion

In our *in vitro* experiment, the biparietal technique results in better tissue penetration, leading to higher transmurality lesions rate. In the uniparietal mode, lesions are broader in size and depth, but they do not reach the same transmurality rate of the first group, suggesting a more random drive of energy through the wall tissue. These findings might be a call for the use of this setting in the clinical practice. Nonetheless, the complexity of the clinical scenario makes this application very challenging, requiring further research to test its real clinical feasibility and applicability.

## Data Availability

The authors declare to have data available.
